# CD4^+^ T Cell Responses to the *Plasmodium falciparum* Erythrocyte Membrane Protein 1 in Children with Mild Malaria

**DOI:** 10.4049/jimmunol.1200547

**Published:** 2014-01-22

**Authors:** Evelyn N. Gitau, James Tuju, Henry Karanja, Liz Stevenson, Pilar Requena, Eva Kimani, Ally Olotu, Domtila Kimani, Kevin Marsh, Peter Bull, Britta C. Urban

**Affiliations:** * KEMRI-Wellcome Trust Collaborative Programme, Centre for Geographic Medicine Coast, 80108 Kilifi, Kenya;; † Liverpool School of Tropical Medicine, Pembroke Place, Liverpool L3 5QA, United Kingdom; and; ‡ Centre for Tropical Medicine, Nuffield Department of Medicine, Oxford University, Oxford OX3 7LJ, United Kingdom

## Abstract

The immune response against the variant surface Ag *Plasmodium falciparum* erythrocyte membrane protein 1 (PfEMP1) is a key component of clinical immunity against malaria. We have investigated the development and maintenance of CD4^+^ T cell responses to a small semiconserved area of the Duffy binding–like domain (DBL)α–domain of PfEMP1, the DBLα-tag. Young children were followed up longitudinally, and parasites and PBMCs were isolated from 35 patients presenting with an acute case of uncomplicated malaria. The DBLα-tag from the PfEMP1 dominantly expressed by the homologous parasite isolate was cloned and expressed as recombinant protein. The recombinant DBLα-tag was used to activate PBMCs collected from each acute episode and from an annual cross-sectional survey performed after the acute malaria episode. In this article, we report that CD4^+^ T cell responses to the homologous DBLα-tag were induced in 75% of the children at the time of the acute episode and in 62% of the children at the following cross-sectional survey on average 235 d later. Furthermore, children who had induced DBLα-tag–specific CD4^+^IL-4^+^ T cells at the acute episode remained episode free for longer than children who induced other types of CD4^+^ T cell responses. These results suggest that a wide range of DBLα-tag–specific CD4^+^ T cell responses were induced in children with mild malaria and, in the case of CD4^+^IL-4^+^ T cell responses, were associated with protection from clinical episodes.

## Introduction

Clinical immunity to malaria requires the induction of both Ag-specific T cell and B cell responses (reviewed in Ref. 1). Ag-specific T cells not only provide T cell help to B cells but also activate the cellular arm of immune responses. One important target of humoral immunity is the *Plasmodium falciparum* erythrocyte membrane protein 1 (PfEMP1), which mediates sequestration of mature forms of the parasite in the vascular bed (2). PfEMP1 is encoded by ∼60 *var* genes per haploid genome that undergo clonal antigenic variation (3). Variants of PfEMP1 mediate adhesion to host receptors such as CD36, ICAM-1, CR1 expressed on endothelial cells, RBCs, and leukocytes, and some variants mediate rosetting of infected RBCs (iRBCs) with uninfected RBCs. Adhesion of mature forms of asexual iRBCs and rosetting in postcapillary venules can lead to obstruction of capillaries with local hypoxia and tissue damage (4).

Recently, *var* genes encoding PfEMP1 from fully sequenced laboratory and clinical parasite isolates have been grouped according to the upstream promoter sequence, chromosomal orientation, and position of *var* genes as well as their adhesion characteristics (5–7). Group A and group B/A PfEMP1 constitute an antigenically restricted subset, and their expression appears to be associated with severe malarial disease (8–15). However, the wide sequence heterogeneity of PfEMP1 variants has rendered analysis of expression patterns on clinical isolates difficult. Bull and colleagues (16) developed a sequence classification system based on a region of the Duffy binding–like domain (DBL)α–domain of PfEMP1, the DBLα-tag, which can be amplified from *var* genes using universal PCR primers and thus is accessible in clinical isolates. The amino acid sequence of amplified DBLα-tags can be grouped according to the number of cysteines (cys2 or cys4), the presence of sequence signatures at “Positions of Limited Variation” (PoLV), and through sharing of a limited number of sequence blocks within the hypervariable regions (17). The majority of group A and group B/A PfEMP1 belong to the group of cys2 PfEMP1. Expression of different subsets of cys2 PfEMP1 has been associated with distinct clinical syndromes and low Ab levels in children suffering from severe malaria (10–13, 16, 18). Clinical immunity to malaria is associated with the accumulation of a wide range of Abs specific for different PfEMP1 variants (12, 19–21). Much less is known about the specificity and phenotype of CD4^+^ T cell responses to PfEMP1, partly because the extreme sequence variability poses a challenge for the analysis of variant-specific T cell responses. Previous studies using recombinant proteins or peptides based on PfEMP1 expressed on laboratory lines showed that individuals living in malaria-endemic areas harbored both IFN-γ− and IL-10–secreting Ag-specific CD4^+^ T cells (22, 23). To identify CD4^+^ T cell responses to PfEMP1 children had encountered during an acute malaria episode, we expressed DBLα-tags representing the dominant PfEMP1 on a parasite isolate and stimulated PBMCs from the child who donated the parasites with this homologous DBLα-tag. Using this system, we showed that DBLα-tag–specific T cells are readily detected in children with acute malaria and maintained for up to 16 wk after an acute episode in a proportion of children (24). The phenotype of CD4^+^ T cell responses to DBLα-tags did not differ between children suffering from severe malaria and those with mild malaria. However, children responding to a homologous cys2 DBLα-tag induced IL-10–secreting CD4^+^ T cells during acute disease but IFN-γ–secreting CD4^+^ T cells 16 wk after an acute malaria episode, suggesting that a stable population of effector memory Th1 cells was maintained.

We wondered whether the phenotype of CD4^+^ T cell responses to DBLα-tags to which a child had been exposed was associated with protection from future malaria episodes. We therefore analyzed CD4^+^ T cell responses to homologous DBLα-tags in a cohort of children under active surveillance for acute malaria episodes. We expressed the homologous DBLα-tag representing the dominant expressed PfEMP1 on infected erythrocytes from 35 children with mild malaria as recombinant proteins. We used the recombinant, homologous DBLα-tag that originated from infected erythrocytes isolated from a given child to investigate the phenotype of Ag-specific CD4^+^ T cells of that child during and after the acute episode. Thus, PBMCs from each child were activated with the recombinant DBLα-tag they had encountered during acute disease. We confirmed that cys2 DBLα-tags induced CD4^+^ T cells that predominantly secrete IL-10 during the acute malaria episode. Of interest, we observed that DBLα-tag–specific CD4^+^IL-4^+^ T cells were associated with delayed time to subsequent malaria episode over a period of 1 y, suggesting a possible role for IL-4–secreting CD4^+^ T cells in protection.

## Materials and Methods

### Study cohort

To test CD4 T cell responses to PfEMP1, parasites and PBMCs were isolated from 35 children under active surveillance for acute malaria selected from a cohort of ∼300 children in Junju, Kilifi District in the Coastal Province of Kenya (25). The inhabitants of Junju are predominantly of the Mijikenda ethnic group and are exposed to biannual peaks of malaria transmission in November–December and May–July. Low-level transmission is also known to occur throughout the year (26). In this population, infection with gastrointestinal helminths can be detected in ∼25% of children but does not alter susceptibility to malaria (27). Acute malaria was defined as an axillary temperature of ≥ 37.5°C and *P.* *falciparum* parasite density of ≥ 2500 parasites per microliter of blood. Children who were acutely ill received medical treatment as required. Between May 2008 and May 2009, 362 children were registered in the cohort, and of these 230 children had at least one acute malaria episode. As part of a larger study, cytoadhesion of infected erythrocytes to CD36 and ICAM-1 was carried out on 95 clinical parasite isolates from children presenting with an acute malaria episode (28). We identified the dominant DBLα-tag representing PfEMP1 expressed on clinical parasite isolates from 50 of these children and were able to express these DBLα-tags as recombinant protein from clinical parasite isolates of 36 children. For 33 children under investigation, PBMCs were collected during the cross-sectional survey in May 2009. Ten of these children had circulating parasites in their blood but did not show any clinical symptoms.

Parents or guardians of the children provided written informed consent. The study was approved by the Kenyan National Ethics Review Committee (SSC no. 1131) and the Oxford Tropical Research Ethics Committee (protocol no. 30-06).

### Processing of blood samples

Blood samples were collected and processed as described previously (24). PBMCs were resuspended in 10% DMSO/FCS and stored in liquid nitrogen, and plasma was stored at −80C. In addition, 100 μl packed RBCs was resuspended in 800 μl TRIzol and stored at −80°C for extraction of RNA.

### Isolation and expression of dominant expressed DBLα-tag

Dominant expressed PfEMP1 were identified by the method described in detail elsewhere (17), with variations described in Ref. 24. In brief, total RNA was extracted from clinical parasite isolates, using TRIzol, and was reversed transcribed to cDNA. The DBLα-tag was amplified with universal primers targeting semiconserved areas of the DBLα-domain of PfEMP1 [DBLαAF′: 5′-GCACG(A/C)AGTTT(C/T)GC-3′; DBLαBR: 5′-GCCCATTC(G/C)TCGAACCA-3′]. The PCR products were cloned into pCR2.1 TOPO cloning vectors, and plasmids from 10 to 20 colonies were isolated. Individual plasmids were sequenced using Big Dye Terminator v3.1 cycle sequencing on an Applied Biosystems 3730 capillary sequencer. The dominant expressed DBLα-tag was identified for each clinical parasite isolate, amplified using the DBLαAF primer and the DBLαBR primer with an additional stop codon, and ligated into the pEXP5(NT) TOPO vector containing an N-terminal His-tag. Plasmids containing the correct sequence were transformed into BL21 DE3pLys *Escherichia coli*, and individual colonies were grown to an OD of 0.4 before induction of protein expression with 1 mM isopropyl β-d-thiogalactoside. Cells were lysed with BugBuster NT containing Benzonase, and recombinant proteins were purified under denaturing conditions using Probond Nickel-Chelating Resin according to the manufacturer’s recommendation. Purified proteins were refolded by dialysis against 20 mM Tris HCl, 50 mM NaCl, and 6 M urea, pH 4.5, with stepwise reduction of the urea concentration over a period of 36 h. Endotoxin was removed using Endotrap Blue according to the manufacturer's recommendation, and endotoxin removal was verified using a *Limulus* amoeba lysate assay. The protein concentration was determined with the BCA assay. Using a classification system previously described, we grouped the recombinant DBLα-tags (European Molecular Biology Laboratory accession numbers HE863905–HE863940; http://www.ebi.ac.uk/embl/) according to the number of cysteines found in their sequence (16).

### Intracellular cytokine staining

PBMCs from acute (36 children) and cross-sectional samples (33 children) were thawed, and 0.5 × 10^6^ cells were seeded two times in triplicate into 96-well plates in medium (RPMI 1698 supplemented with 5% pooled human AB serum, 5 mM glutamine, 10 mM HEPES, 50 μM 2-MΕ, and 50 μM kanamycin). Cells were rested overnight before activation with medium alone, 20 μg/ml homologous recombinant DBLα-tag (isolated from infected erythrocytes with which the child was infected during acute disease), or anti-CD2/CD3/anti-CD28–coated MACSiBead Particles (Miltenyi Biotec) in the presence of 1 μg/ml CD28 and CD49d for 2 h. Cells were incubated for another 18 h in the presence of brefeldin A. Cells were harvested and stained with ViViD Aqua (Invitrogen) before intracellular cytokine staining was performed as follows: Cells were fixed with Cytofix (Becton Dickinson) for 20 min at room temperature in the dark, washed twice with Cytoperm (Becton Dickinson), and subsequently stained in Cytoperm with CD3-ECD (Beckman Coulter), CD4-PerCP (Becton Dickinson), CD8-APC H7 (Becton Dickinson), IL-2–FITC (Becton Dickinson), IL-10–PE (Becton Dickinson), IFN-γ–PECy7 (Becton Dickinson), and IL-4–APC (Becton Dickinson), for 1 h at 4°C in the dark. Cells were washed twice and resuspended in Sheath Fluid (Beckman Coulter), and ≥ 100,000 lymphocytes were acquired on a Cyan Analyzer (Beckman Coulter) within 24 h. Single stained BD comp beads were processed and acquired in parallel to PBMC samples each day and used to set postacquisition compensation in FlowJo. Live CD3^+^CD4^+^ or CD3^+^CD8^+^ T cells were identified, and the proportion of intracellular cytokine-producing T cells was determined using FlowJo Africa. Samples with < 1000 CD4^+^ T cells (*n* = 1 acute episode and *n* = May 7, 2009 cross-sectional survey) were excluded from further analysis. Thus, data presented in this article were available for 35 children with acute malaria and 26 children during the cross-sectional survey in May 2009. The median CD4^+^ T cell count acquired in the remaining samples was 50,760 CD4^+^ T cells (range: 2321–230,000). Values obtained from PBMCs incubated with medium alone were subtracted from values obtained after activation of PBMCs with DBLα-tags. When individual gates of cytokine-secreting CD4^+^ T cells had fewer than five positive events or their percentage was <0.03%, the response was recorded as zero. The gating strategy for one representative sample is shown in Supplemental Fig. 1. Because the background of CD4^+^IL-4^+^ T cell responses was high in medium control samples of some children, we tested the specificity of our IL-4 Ab. When we replaced the anti–IL-4–APC Ab with an isotype control Ab or blocked IL-4 with an unconjugated anti–IL-4 Ab, almost no background staining was observed (Supplemental Fig. 2).

### ELISA

Plasma cytokine concentrations for TNF-α, IL-12, IL-10, TGF-β, IFN-γ, and IL-4 were determined using commercially available ELISA kits (R&D Systems, Minneapolis, MN, TNF-α and BD Sciences, San Jose, CA, for all other cytokines). The ELISAs were performed according to the manufacturers’ instructions. The detection limits for all cytokines was at or below 20 pg/ml.

For Ab reactivity in sera against DBLα-tags, ELISA plates (MaxiSorp; Nunc) were coated with 1 μg/ml individual DBLα-tags diluted in PBS overnight at 4°C. Plates were washed three times with PBS and then blocked with 3% BSA (Sigma-Aldrich) diluted in PBS and 0.01% Tween 20 (VWR) for 2 h at room temperature. Plates were washed three times with PBS and 0.01% Tween 20 and incubated in duplicate with a 1:200 dilution of individual patient serum diluted in 3% BSA, PBS, and 0.01% Tween 20 for 2 h at room temperature. Each DBLα-tag was also tested against sera from five European donors not exposed to malaria and five hyperimmune donors. Plates were washed three times, as before, and subsequently incubated with rabbit anti-human IgG conjugated to HRP (DAKO Cytomation) diluted 1:5000, anti-human IgG4 (Southern Biotech), or anti-human IgE (Southern Biotech), 1:125 in 3% BSA, PBS, and 0.01% Tween 20 for 1 h at room temperature. Plates were washed three times, as before, and developed with OPD (Sigma-Aldrich) for 20–30 min, and the OD was measured at 490 nm.

### Statistical data analysis

Mean responses were calculated, and a Student *t* test was conducted for normally distributed variables. Median responses were calculated, and the Wilcoxon rank sum test was conducted for variables with a nonnormal distributions. Logistic regression was used to examine independent relationships of dominant expression of cys2- and cys4-PfEMP1 on a given clinical isolate, and the phenotype of the CD4^+^ T cell response at the acute episode was adjusted for both age and parasite density. To analyze whether the phenotype of the CD4^+^ T cell response at the acute episode conferred protection over 1-y of follow-up, Cox regression with the primary endpoint of time to next episode of clinical malaria after the acute event was used and adjusted for age at time of acute illness. Stata version 11.2 and R version 2.11.1 were used for statistical analysis. Graphs were generated using Stata version 11.2 and GraphPad Prism version 5.0.

## Results

### Phenotype of T cell responses to PfEMP1 during the acute malaria episode

**FIGURE 1.**
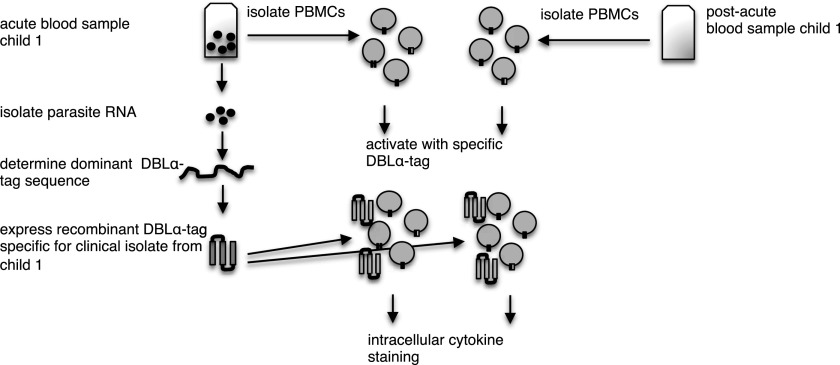
Schematic presentation of the study design. From each child, blood samples were collected at the acute malaria episode or during the cross-sectional survey (postacute). PBMCs and plasma were separated and stored. RBCs were separated from the acute blood sample and total parasite RNA isolated using Trizol. Total RNA was reversed transcribed and DBLα-tag sequences amplified by PCR. DBLα-tag PCR products were cloned and 10–20 clones sequenced. The dominant DBLα-tag sequence was cloned into an expression vector and the recombinant DBLα-tag purified. The recombinant DBLα-tag originating from PfEMP1 expressed on the clinical parasite isolate a child was infected with (the homologous DBLα-tag) was used to stimulate stored PBMCs from that child and CD4^+^ T cell responses were analyzed by intracellular cytokine staining and flow cytometry.

**FIGURE 2.**
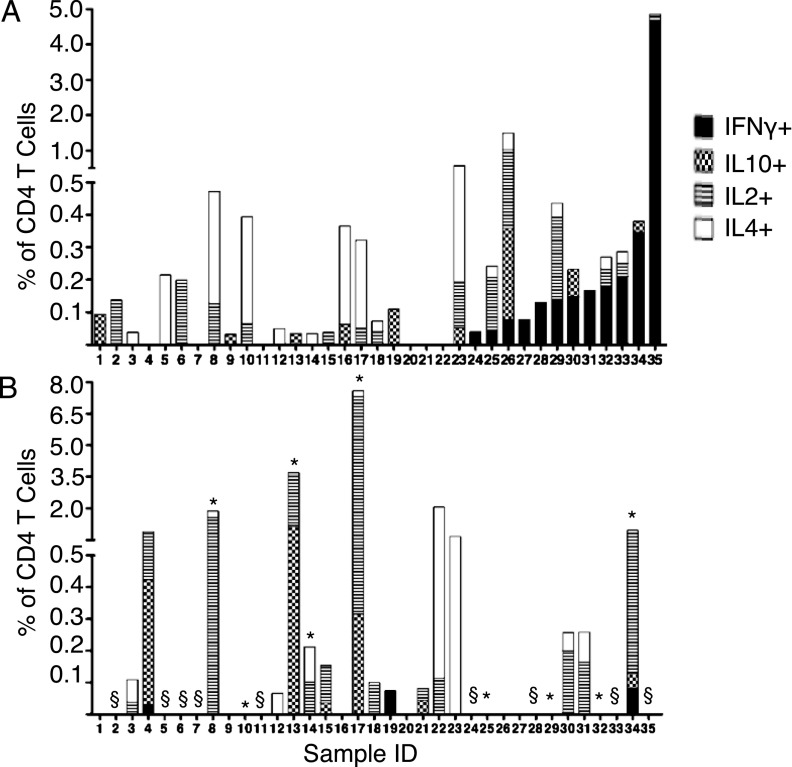
Cumulative percentage of cytokine producing CD4^+^ T cells after stimulation with homologous DBLα-tag. Shown are cumulative bar-graphs of the percentage of DBLα-tag specific CD4^+^ T cells producing cytokines as indicated for each child at the acute episode (**A**) and at the cross-sectional survey (**B**). Children with asymptomatic parasitaemia at the cross-sectional survey are indicted (*). Children with missing data at the cross-sectional survey (*n* = 9) are also indicated (§).

We analyzed CD4 T cell responses to the recombinant homologous DBLα-tags representing the dominant expressed PfEMP1 on each clinical isolate from 35 children presenting with an acute episode between May 2008 and May 2009 (a schematic overview is presented in Fig. 1). Overall, cytokine-secreting, Ag-specific CD4^+^ T cells were detected in 27 (75%) children in response to the DBLα-tag at the acute episode (Fig. 2). There were no significant differences in either parasite densities, age, WBC counts, eosinophil counts or hemoglobin levels in children with or without Ag-specific CD4^+^ T cell responses at the time of acute disease (Table I). Although infection with gastrointestinal helminths was not measured but is known to be prevalent in this area, none of the children showed mild eosinophilia (median (25th and 75th percentile): 0.12 × 10^3^/μl (0.06–0.15 × 10^3^/μl) suggesting that acute infection with gastrointestinal helminths can be excluded. In addition, we measured the concentration of proinflammatory and anti-inflammatory cytokines in plasma (Supplemental Fig. 3). The number of children with detectable levels of TNF-α (*n* = 4) and IL-4 (*n* = 5) was low. Overall, cytokine levels did not differ significantly between those children who showed Ag-specific CD4^+^ T cell responses and those who did not.

**Table I.** Baseline parameters of children inducing T cell responses at the acute episode

	Responders	Non-Responders	
	*n* = 27	*n* = 8	*p*
Age at acute event (months)	66.5 (49.6–84.8)	68 (43.2–90.2)	0.783
Parasite density (iRBCx10^3^/μl)	150 (73–222)	128 (19.3–232)	0.738
RBC (10^9^/μl)	4.4 (4.1–4.8)	4.5 (4.4–4.8)	0.651
Hb (g/dl)	10.4 (9.6–10.9)	10.9 (10.4–12.6)	0.116
WBC (10^6^/μl)	8.5 (6.9–11.4)	8.2 (7.6,9.1)	0.898
Eosinophils (10^3^/μl)	0.13 (0.06–0.19)	0.09 (0.06–0.14)	0.508
Time to next episode (days)	249 (194.5–356)	199 (62.5–303.2)	0.276
TNFα (pg/ml)	0 (0,0)	0 (0–5.5)	0.159
IL-12 (pg/ml)	26.8 (0–115.9)	96.2 (9.6–198.4)	0.278
IL-10 (pg/ml)	726.6 (211–1666)	1041 (332–2355)	0.555
TGF-β (pg/ml)	639.9 (0–4008)	190.7 (0–3647)	0.638
IFN-γ (pg/ml)	0 (0–38.8)	9.3 (0–22.3)	0.842
IL-4 (pg/ml)	0 (0–0)	0 (0–0)	0.208

Shown are median and 25th and 75th percentile. Differences between the groups were determined by Mann–Whitney *U* test.

**FIGURE 3.**
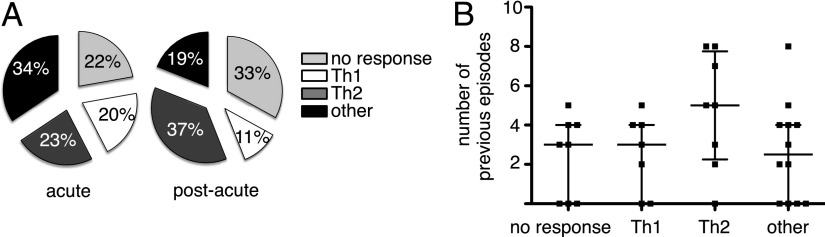
Phenotype of CD4^+^ T cells stimulated with the homologous DBLα-tag. The phenotype of CD4^+^ T cell in response to the homologous DBLα-tag was classified for each child as follows: Th1: IFN-γ– but no IL-4–secreting CD4^+^ T cells; Th2: IL-4– but no IFN-γ–secreting CD4 T cells; Other: All other responses including mixed Th1/Th2 responses; No responses: children who did not induce any Ag-specific CD4^+^ T cells. (**A**) Shown are pie charts of the proportion of all children who induced a given CD4^+^ T cell phenotype in response to the DBLα-tag at the acute episode (*n* = 35) and at the cross-sectional survey in May 2009 (*n* = 26). (**B**) Number of episodes before the acute event in children with different CD4^+^ T cell phenotypes. Shown are median (horizontal bar) and 25th and 75th percentile (vertical bars). The differences in the number of episodes in children with a dominant Th2 response compared with the number of episodes in children with other responses were not significant.

During acute disease, 12 (34.3%) children had DBLα-tag specific IFN-γ^+^ CD4^+^ T cells either alone or in the presence of CD4^+^ T cells secreting other cytokines. Of these 12 children, 5 also induced DBLα-tag specific IL-10^+^ CD4^+^ T cells and 2 children induced IFN-γ^+^IL-10^+^ double producing CD4^+^ T cells. To define the phenotype of DBLα-tag specific CD4^+^ T cells, responses were classified as Th1, Th2 or “other” responses. CD4^+^ T cell responses in children with IFN-γ– but no IL-4–secreting CD4^+^ T cells were defined as a Th1 response and CD4^+^ T cell responses in children with IL-4– but no IFN-γ–secreting T cells were defined as Th2 response. All other responses including a mixed Th1/Th2 response were defined as “other” (Fig. 3A). There was no difference in the proportion of children with either a dominant Th1 or Th2 response to the DBLα-tag at the acute malaria episode. There were no significant differences between the number of children inducing Th1 and Th2 responses with respect to parasite density or age at the time of the acute episode. Children who induced a Th2 response had a higher number of prior malaria episodes during their lifetime compared with children who induced Th1 responses, a mixed response or no response although the differences were not significant (Fig. 3B). As expected, there was a significant difference in the median IFN-γ plasma cytokine levels (median [25th–75th percentile]: Th1 = 41.9pg/ml [10–105], Th2 = 0 pg/ml [0–0], Mann–Whitney *p* = 0.0124) between children who induced a Th1 CD4 T cell response and those that did not. There was no significant difference in the mean IL-4 plasma cytokine concentration between children who induced a dominant Th1 response (median [25th–75th percentile]: 0 pg/ml [0–0]) and those that induced a Th2 response (median [25th–75th percentile]: 0 pg/ml [0–0]) due to the small number of children with detectable levels of plasma IL-4.

### Phenotype of T cell responses to PfEMP1 after the acute malaria episode

We analyzed CD4^+^ T cell recall responses to the DBLα-tag representing the dominant PfEMP1 expressed on the clinical isolate during acute disease in 26 children from whom PBMCs had been collected during the cross-sectional survey in May 2009 (Fig. 2B). At the cross-sectional survey all but 9 children were free of parasites. Parasite positive children were included in the analysis because they did not present with any clinical symptoms and showed a similar profile of CD4^+^ T cell responses to the homologous DBLα-tag as parasite-free children (Supplemental Table I). The average number of days between the acute event and collection of blood samples during the cross-sectional survey was 235 d (CI 204–265). During the cross-sectional survey, 62% (16/26) children mounted DBLα-tag specific recall CD4^+^ T cell responses. Interestingly, children who showed any DBLα-tag specific CD4^+^ T cell responses had lower parasite densities at the acute episode than non-responders (Table II). This was true for the 11 children who displayed DBLα-tag specific IL-4–secreting CD4^+^ T cells (t = 2.83 *p* = 0.011) and the 14 children inducing IL-2^+^–secreting CD4^+^ T cells (*t* test = 2.16 *p* = 0.040) but not for those who induced IL-10– or IFN-γ–secreting CD4^+^ T cells (*n* = 5 and *n* = 1, respectively).

**Table II.** Comparison of baseline parameters in children with or without DBLα-tag specific CD4 T cell responses at the cross-sectional survey

	Responder	Non-Responder	
	*n* = 16	*n* = 10	*p*
Age at survey (months)	77.5 (60.7–96.7)	79.2(71.8–101.8)	0.527
Parasite density (iRBC × 10^3^/μl) acute	76.8 (16.2–129.1)	207.9 (169.5–293.2)	**0.008**
Parasite density (iRBC × 10^3^/μl) survey*	0 (0–0.54)	0 (0–19)	0.804
RBC (10^6^/μl)	4.6 (4–4.8)	4.8 (4.6–4.9)	0.282
Hb (g/dL)	11.3 (11.1–11.8)	12.6 (12.5–12.7)	**0.05**
WBC (10^6^ /μl)	8.2 (5.9–10.3)	5.7 (5.2–7)	0.09
Time from acute event (days)	267 (169–291)	276 (246–293)	0.477

Shown are median and 25th and 75th percentile. Differences between the groups were determined by Mann–Whitney *U* test. Bold indicates statistically significant *p* values <0.05.

*Nine children had low-level parasite densities (six responders and three non-responders). However, their data were not excluded as they were all asymptomatic with a temperature of < 37.5°C.

When we compared the phenotypes of CD4^+^ T cell responses induced during the acute episode with those induced during the cross-sectional survey, we observed no significant differences in the overall profile (Fig. 3A). However, the proportion of children with DBLα-tag specific IFN-γ–secreting CD4^+^ T cells significantly dropped (Fisher’s exact test: acute *n* = 12, postacute *n* = 3, *p* = 0.0028).

### CD4^+^ T cell responses during acute disease are associated with specific subgroups of PfEMP1

**FIGURE 4.**
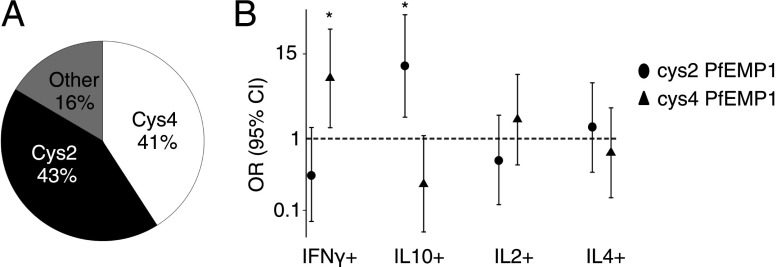
Relationship between CD4^+^ T cell responses to cys2 and cys4 PfEMP1. (**A**) Pie chart representing the proportion of recombinant DBLα-tags falling into cys2 (PoLV group1-3), cys4 (PoLV group 4 and 5) or none (PoLV group 6) of these groups (16). (**B**) Odds ratios and 95% confidence intervals are plotted for CD4^+^ T cells secreting a cytokine as indicated when children were infected parasites dominantly expressing either cys2 PfEMP1 (circle) or cys4 PfEMP1 (triangle). Children infected with cys2 PfEMP1-expressing iRBCs were more likely to induce CD4^+^IL10^+^ responses whereas children infected with cys4 PfEMP1-expressing iRBC were more likely to induce CD4^+^IFN-γ^+^ T cell responses. *p < 0.05.

We analyzed whether CD4^+^ T cell responses to the DBLα-tag were associated with a specific subgroup of PfEMP1 as previously observed (24). DBLα-tags were grouped into those containing 2 cyteines (cys2) and those containing 4 cysteines (cys4) (Fig. 4A). Children harboring iRBCs dominantly expressing cys2 PfEMP1 (15/35) were more likely to induce IL-10–secreting CD4^+^ T cells (*n* = 10, Odds Ratio 10.2 *p* = 0.006) than children harboring iRBCs expressing non-cys2 PfEMP1 (Fig. 4B). By contrast, children harboring iRBCs dominantly expressing cys4 PfEMP1 (17/35) were more likely to induce CD4^+^ IFN-γ^+^ T cells at the acute episode (*n* = 9, Odds Ratio 6.8 *p* = 0.017) compared with children harboring iRBCs expressing non-cys4 PfEMP1. In line with the observations above, children infected with parasites dominantly expressing a cys4 PfEMP1 had higher median proportion of CD4^+^IFN-γ^+^ T cells whereas those infected with parasites dominantly expressing a cys2 PfEMP1 had a higher median proportion of CD4^+^IL-10^+^ T cells (Table III).

**Table III.** Comparison of CD4 T cell responses to cys2 and cys4 PFEMP1

	Cys2	Cys4	*p*
CD4^+^IFN-γ^+^ (%)	0 (0–0)	0.04 (0–0.173)	**0.027**
CD4^+^IL-10^+^ (%)	0.033 (0–0.08)	0 (0–0)	**0.0021**
CD4^+^IL2^+^ (%)	0 (0–0.06)	0.04 (0–0.14)	0.324
CD4^+^IL-4^+^ (%)	0 (0–0.32)	0.034 (0–0.038)	0.699

Shown are median 25th and 75th percentile. Differences in the percentage of CD4^+^ T cells secreting cytokines as indicated were determined by Mann–Whitney *U* test. Bold indicates statistically significant *p* value <0.05.

### DBLα-tag specific IL-4–secreting CD4^+^ T cells are associated with protection

We wondered whether either the phenotype of a CD4^+^ T cell response or the PfEMP1 subgroup dominantly expressed by the clinical isolate a child was infected with correlated with time to subsequent malaria episode a child had over the next year thus indicating association of a particular response with protection.

**FIGURE 5.**
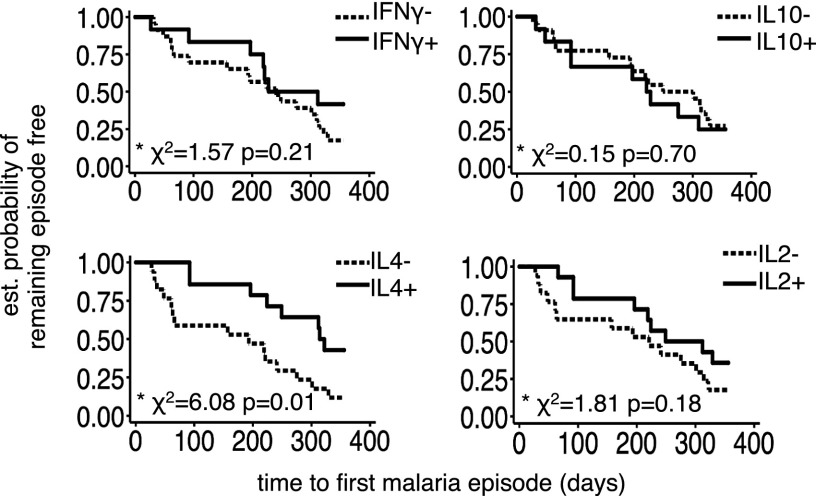
Survival plots with time to first episode of clinical malaria over a 12 mo period. Survival plots for children who induced DBLα-tag specific CD4^+^ T cells secreting a given cytokine. Children who made a CD4^+^IL-4^+^ T cell response (*n* = 14) remained episode free longer than children who did not (HR = 0.31, CI 0.12–0.79, *p* = 0.014, adjusted for age). Hazard ratios for all other types of T cell responses were not significant. *Log rank test for equality.

Cox regression (adjusted for age) showed that those children who induced DBLα-tag specific IL-4^+^ CD4^+^ T cell responses during the acute episode remained episode-free for longer (HR = 0.31, CI 0.12–0.79, *p* = 0.014; test of proportional hazards assumption χ^2^ = 15.39 *p* = 0.0005) than children who induced DBLα-tag specific CD4^+^ T cells secreting either IFN-γ, IL-2 or IL-10 (HR = 0.56, CI 0.21–1.47, *p* = 0.243; HR = 0.51, CI 0.20–1.29, *p* = 0.152; HR = 1.17, CI 0.517–2.66, *p* = 0.702) (Fig. 5). Of note, the majority of children who induced Th2 responses to the DBLα-tag during the cross-sectional survey (8/26) remained episode free for at least 7 mo after the acute event. In addition, children who induced Th2 responses (*n* = 8) during acute malaria tended to have higher number of total malaria episodes prior to the acute event (Fig. 3B) than children who induced a Th1 response (odds = 1.45 *p* = 0.148; adjusted for age at acute illness) although the difference was not significant. Thus, induction and maintenance of Ag-specific IL-4^+^ CD4^+^ T cell responses are associated with protection independent of the subgroup of PfEMP1 expressed on the acute isolate.

### Ab responses to the homologous DBLα-tag

**FIGURE 6.**
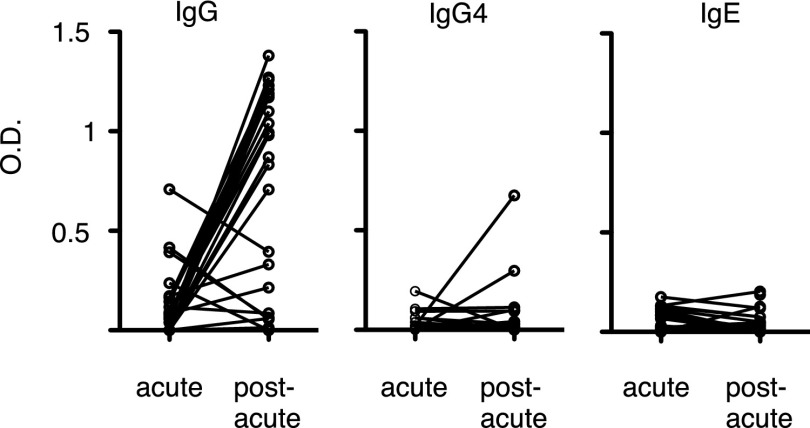
Ab responses to the homologous DBLα-tag at the acute episode and the cross-sectional survey. Shown are corrected OD values of DBLα-tag specific Ab responses for each child at the time of acute disease and at the cross-sectional survey for IgG, Ig4, and IgE.

We determined the induction of IgG Abs to the DBLα-tag representing the dominant expressed PfEMP1 on iRBCs isolated at acute disease in each child by ELISA. In addition, we analyzed the induction of IgG4 and IgE Abs to the DBLα-tag because the Ab isotypes are associated with a classical Th2 response. The DBLα-tag specific IgG Ab concentration increased from a median OD value of 0 (25th and 75th percentile: 0–0.12) at acute disease to a median OD value of 0.7 (25th and 75th percentile: 0.1–1.17) at the cross-sectional survey. In total, 18 out of 31 children induced IgG Abs to the homologous DBLα-tag between acute disease and the cross-sectional survey whereas 13 children either did not induce or lost their existing Ab response (Fig. 6). DBLα-tag specific IgG4 and IgE Abs were low or not detectable in all children (postacute median OD value [25th and 75th percentile] for IgG4: 0.0 [0–0.02], for IgE: 0.0 [0–0.047]). Two children induced DBLα-tag specific IgG4 responses after the acute malaria episode but neither child had induced IL-4–secreting CD4^+^ T cells. Only in children who gained an IgG Ab response after the acute malaria episode, we observed a weak positive correlation between the percentage of IL-10–producing CD4^+^ T cells at acute disease and induction of DBLα-tag specific Ab responses to the homologous DBLα-tag (Spearman rho = 0.579, *p* = 0.0118) but not with other cytokine-producing CD4^+^ T cells. IL-10 is a potent cytokine for the induction of long-lived plasma cells, which may explain the association between IL-10–secreting CD4^+ ^T cells and induction of Ab responses to the homologous DBLα-tag.

## Discussion

The sequence diversity of PfEMP1 makes it difficult to analyze immune responses to variants of this Ag expressed on clinical isolates. Only a small region of the DBLα-domain of PfEMP1, the DBLα-tag, can be targeted with universal primers amplifying almost all variants identified to date (16). We recently reported that almost all children attending Kilifi District Hospital with acute malaria induced CD4^+^ T cell responses to recombinant DBLα-tags that were identified in the clinical isolate they were infected with. Using the same system, we investigated the phenotype of CD4^+^ T cells specific for the DBLα-tag identified in each clinical isolate for 35 children suffering from acute malaria and who were under active surveillance for acute malaria episodes. We show in this article that children who induced DBLα-tag specific IL-4^+^CD4^+^ T cell responses had a delayed time to subsequent malaria episodes over the next year. These data suggest that DBLα-tag specific CD4^+^IL-4^+^ T cells contribute to protection from future malaria episodes.

Almost all children induced DBLα-tag specific CD4^+^ T cells secreting any of the cytokines measured during the acute malaria episode and 62% maintained effector cells for over 6 mo suggesting that effector memory T cells had been induced. Interestingly, the proportion of children who maintained Ag-specific IFN-γ–secreting CD4^+^ T cells had dropped significantly at the cross-sectional survey. We confirmed our previous observation (24) that children suffering from acute malaria and infected with clinical isolates that dominantly expressed cys2 PfEMP1 were more likely to induce IL-10–secreting CD4^+^ T cells during acute disease. By contrast, children infected with clinical isolates dominantly expressing cys4 PfEMP1 were more likely to induce IFN-γ–secreting CD4^+^ T cells. We did not observe the later association in children attending Kilifi District Hospital with acute malaria even though the majority of these children did not suffer from severe, life-threatening malaria (24). The reasons for this discrepancy are unknown but children attending the Kilifi District Hospital tended to be younger and showed higher parasite density. It is possible that they were more severely ill due to later detection of parasites or that they failed to mount an adequate immune response to cys4 PfEMP1 or both. In humans, several studies reported an association of IFN-γ production by CD4^+^ T cells but also NK and γδ T cells with protection from symptomatic malaria (reviewed in Ref. 29). Furthermore, experimentally infected volunteers and Thai adults living in a low-transmission area maintained IFN-γ–secreting effector memory T cells for at least a year (30, 31). However, other studies indicated that downregulation of IFN-γ responses occurred in individuals repeatedly exposed to malaria (32–34) but re-emerged when previously immune individuals were no longer exposed (35). Together these data suggest that IFN-γ–production by Th1 cells and other lymphocytes may be important for control of parasitaemia in individuals with low immunity for instance through activation of cellular responses. With increasing exposure and acquisition of Ab responses to a range of malarial Ags, efficient T cell help for B cells might be more important for clinical immunity to malaria.

In this study, we observed a clear association of IL-4–producing T cells specific for DBLα-tags with prolonged time to reinfection and clinical malaria. Interestingly, children who maintained IL-4–producing responses to the DBLα-tag tended to have a higher rate of past exposure but lower parasitaemia during the acute disease than those children inducing other T cell responses. Studies in animal models of malaria demonstrated that protection from malaria is dependent on both Th1 and Th2 responses. In these models, early induction of IFN-γ–producing CD4^+^ T cells was required for the control of parasitaemia through activation of innate immune responses whereas clearance of parasites was dependent on the induction of Th2 T cells and the presence of B cells (36, 37). Studies conducted in humans demonstrated that Ag-specific IL-4^+^ producing T cells were associated with elevated levels of malaria-specific serum IgG (38) and that Th1 cytokines were dominant at acute disease followed by a Th2 response during parasite clearance (39, 40). The proportion of individuals with IL-4–producing lymphocytes in responses to parasite Ag was higher among the Fulani, who are more resistant to malaria, than the sympatric group of Dogon (41). In addition, Th2 responses to malarial Ags have been studied in the context of polymorphisms in the IL-4 promoter region affecting the production of IL-4. Several studies showed that polymorphism in the IL-4 promoter region were associated with either protection or susceptibility to severe disease in populations living under different transmission intensities (42–44). In addition, Th2-dependent induction of Ag-specific IgE has been reported in some studies and associated with both protection (45) and susceptibility to malaria (46). In summary, although the relative importance of Th2 responses in human malaria remains somewhat elusive the available evidence suggests that both late during a single infection as well as with repeated infection, IL-4–secreting T cells increase whereas IFN-γ–producing CD4^+^ T cells decrease (47) as has been shown in mouse models of malaria.

T cells provide help to B cells through differential expression of costimulatory molecules thus driving activation and differentiation of B cells, but also require reciprocal activation by B cells for differentiation. The main role of CD4^+^ Th cells in tissues and peripheral circulation is probably to activate innate cellular responses in a pathogen specific manner whereas follicular helper T cells (Tfh) producing IL-21 are now considered the main T cell subset providing help to B cell (reviewed in Ref. 48). Both, Tfh and Th2 cells can produce IL-4 and thus IL-4 production by T cells can occur in a Th2-independent manner and, in line with the increasing recognition of T cell plasticity, exert different functions dependent on location and cellular context (49).

IL-21 and IL-4 have largely overlapping roles in the induction of the germinal center reaction and Ab responses. More specifically IL-4 is important for somatic hypermutation, class switching as it promotes isotype switching to all IgG subclasses (50, 51) and induction of memory B cell differentiation (52). By contrast, IL-21 and, to a lesser extent, IL-10 is critical for the induction and survival of plasma cells (53–55).

To establish whether DBLα-tag specific CD4^+^IL-4^+^ were associated with induction of Ag-specific Abs, we measured DBLα-tag specific total IgG as well as IgG4 and IgE in plasma from acute and cross-sectional samples. We did not observe a correlation between IL-4–secreting T cells and DBLα-tag-specific Abs in plasma at the time of the cross-sectional survey for any of the Ab subclasses measured.

Therefore, at least in our study, IL-4 secretion by Ag-specific T cells was not associated with increased plasma concentration of DBLα-tag-specific Abs. It has been reported recently that PBMCs from malaria immune donors produced IL-21 and that increased plasma concentration of IL-21 correlated with Ag-specific IgG1 and IgG3 concentrations and (56, 57). Given the differential role of IL-4 and IL-21 for memory or plasma cell differentiation respectively, it seems likely that IL-4–secreting CD4^+^ T cells promoted the induction of DBLα-tag specific memory B cells rather than long-lived plasma cells. Indeed, it has been repeatedly reported that in children, Abs to malarial Ags rapidly decline and are maintained only in the presence of parasites (58–60) whereas memory B cells are induced and maintained even in the absence of exposure for several years (61, 62). Further studies analyzing the relationship between Ag-specific memory B cells, Ab levels and different CD4^+^ T cell subsets including Th2 and Tfh T cells are required to elucidate these interactions and their relevance for clinical immunity to malaria in humans.
